# Mapping myelin in white matter with T1-weighted/T2-weighted maps: discrepancy with histology and other myelin MRI measures

**DOI:** 10.1007/s00429-022-02600-z

**Published:** 2023-01-24

**Authors:** Stefano Sandrone, Marco Aiello, Carlo Cavaliere, Michel Thiebaut de Schotten, Katja Reimann, Claire Troakes, Istvan Bodi, Luis Lacerda, Serena Monti, Declan Murphy, Stefan Geyer, Marco Catani, Flavio Dell’Acqua

**Affiliations:** 1 Department of Brain Sciences, Faculty of Medicine, Imperial College London, London, UK; 2 NatBrainLab, Department of Forensic and Neurodevelopmental Sciences, Institute of Psychiatry, Psychology & Neuroscience, King’s College London, London, UK; 3 The Sackler Institute for Translational Neurodevelopment, Institute of Psychiatry, Psychology & Neuroscience, King’s College London, London, UK; 4 IRCCS SYNLAB SDN S.p.A., Naples, Italy; 5 Brain Connectivity and Behaviour Laboratory, Sorbonne Universities, Paris, France; 6 Groupe d’Imagerie Neurofonctionnelle, Institut des Maladies Neurodégénératives-UMR 5293, CNRS, CEA University of Bordeaux, Bordeaux, France; 7 Department of Neurophysics, Max Planck Institute for Human Cognitive and Brain Sciences, Leipzig, Germany; 8 Paul Flechsig Institute of Brain Research, University of Leipzig, Leipzig, Germany; 9 Department of Basic and Clinical Neuroscience, Institute of Psychiatry, Psychology & Neuroscience, King’s College London, London, UK; 10 Clinical Neuropathology, King’s College Hospital NHS Foundation Trust, London, UK; 11 Department of Neuroimaging, Institute of Psychiatry, Psychology & Neuroscience, King’s College London, London, UK; 12 Developmental Imaging and Biophysics Section, UCL Great Ormond Street Institute of Child Health, London, UK; 13 Institute of Biostructures and Bioimaging, National Research Council, Naples, Italy

**Keywords:** Corpus callosum, Myelin mapping, Neuroanatomy, Neuroimaging, Validation, Quantitative susceptibility mapping, Myelin water fraction

## Abstract

The ratio of T1-weighted/T2-weighted magnetic resonance images (T1w/T2w MRI) has been successfully applied at the cortical level since 2011 and is now one of the most used myelin mapping methods. However, no reports have explored the histological validity of T1w/T2w myelin mapping in white matter. Here we compare T1w/T2w with ex vivo postmortem histology and in vivo MRI methods, namely quantitative susceptibility mapping (QSM) and multi-echo T2 myelin water fraction (MWF) mapping techniques. We report a discrepancy between T1w/T2w myelin maps of the human corpus callosum and the histology and analyse the putative causes behind such discrepancy. T1w/T2w does not positively correlate with Luxol Fast Blue (LFB)-Optical Density but shows a weak to moderate, yet significant, negative correlation. On the contrary, MWF is strongly and positively correlated with LFB, whereas T1w/T2w and MWF maps are weakly negatively correlated. The discrepancy between T1w/T2w MRI maps, MWF and histological myelin maps suggests caution in using T1w/T2w as a white matter mapping method at the callosal level. While T1w/T2w imaging may correlate with myelin content at the cortical level, it is not a specific method to map myelin density in white matter.

**Supplementary Information:**

The online version contains supplementary material available at 10.1007/s00429-022-02600-z.

## Introduction

Half of the human brain is composed of white matter tissue, which primarily contains myelinated axons of various diameters (Alberts et al. 2014; Quarles et al. 2006). Myelin sheath surrounds axons and is essential for the efficient conduction of action potential between neurons (Nieuwenhuys et al. 2007; Kandel et al. 2012). Several human brain diseases are associated with white matter pathology (Lazzarini 2003; Schmahmann and Pandya 2006; van der Knaap and Valk 2011; Catani and Thiebaut de Schotten 2012). It is, therefore, crucial to have reliable and valid methods to map brain myelination in the living brain.

Advances in structural imaging include approaches towards myelin mapping, such as the ratio of T1-weighted/T2-weighted Magnetic Resonance Imaging (MRI) scans (Glasser and Van Essen 2011; Glasser et al. 2014), multi echo-T2 (MacKay et al. 1994; Zhang et al. 2015), T1 maps (Geyer et al. 2011; Geyer and Turner 2013; Bock et al. 2013), mcDespot myelin water fraction (Deoni et al. 2008; Deoni and Kolind 2015), macromolecular tissue volume (Mezer et al. 2013) and magnetization transfer (MT) methods (Hagiwara et al. 2018; Henkelman et al. 2001; Stanisz et al. 1999).

Myelin is present in the cortex, but it is most abundant in the cerebral white matter (Shafee et al. 2015), and the ratio of T1-weighted/T2-weighted (T1w/T2w) MRI images has been suggested as a reliable and straightforward in vivo method to map myelin in the cerebral cortex (Glasser and Van Essen, 2011). Some studies have even applied T1w/T2w to white matter tracts in the neonatal (Lee et al. 2015; Chen et al. 2017) and adult brain (Colmenares et al. 2021). However, upon comparison with other myelin mapping imaging modalities, including Myelin Water Fraction (MWF), T1w/T2w ‘may be not an optimal index of subcortical myelin content’ (Arshad et al. 2017), and its use as a myelin marker has been criticized (Uddin et al. 2018, 2019). Currently, there is still no histological evidence of the validity of T1w/T2w as a myelin mapping method when applied to white matter at the subcortical level.

This study, therefore, aims to clarify whether in vivo T1w/T2w ratio is a method for mapping myelin density in white matter with (i) histological and (ii) imaging evidence. Specifically, we looked at in vivo T1w/T2w myelin mapping of the corpus callosum from 67 healthy subjects from the Human Connectome Project (HCP) and we acquired ex vivo myelin staining from four post-mortem callosal samples. Additional quantitative susceptibility mapping (QSM) (Li et al. 2015) and MWF data (MacKay et al. 1994; Zhang et al. 2015) were included to complement the MRI analysis.

We focused on the corpus callosum because it represents, among all the brain pathways, not only the largest white matter tract, but also the most thoroughly examined using in vivo and post-mortem techniques in animals and humans (Zaidel and Iacoboni 2003). A further advantage is that an established geometric subdivision of the corpus callosum is thought to reflect different cortical projections (Witelson 1989). According to this subdivision, the rostrum and the genu project to the prefrontal cortex, whereas the posterior body and the splenium project to the sensory-motor and visual areas, respectively (Zaidel and Iacoboni 2003; Catani and Thiebaut de Schotten 2012). Microscopy studies of the corpus callosum demonstrated a heterogeneous distribution of fibres along the antero-posterior axis (Aboitiz et al. 1992; Zaidel and Iacoboni 2003; Caminiti et al. 2009, 2013). Unmyelinated fibres make up 16% of the genu and under 5% of other callosal areas; moreover, the posterior midbody is the region with the highest density of large and highly myelinated callosal fibres (Aboitiz et al. 1992). This permits us to put forward specific hypotheses about the topographical distribution of the myelin density along the corpus callosum. Hence, our primary goal is to demonstrate a converging myelin pattern of the corpus callosum using T1w/T2w MRI and ex vivo post-mortem histology. Considering that myelin and iron distributions overlap significantly in several brain regions (Ogg and Steen 1998; Fukunaga et al. 2010), additional QSM MRI data were acquired on healthy subjects to test whether T1w/T2w callosal maps are, instead, modulated by, or might reflect, iron density. Finally, a recently released MWF atlas of the healthy human brain based on multi-echo T2 MRI (Liu et al. 2019) has allowed a direct comparison between the results obtained with T1w/T2w and an alternative in vivo MRI myelin mapping technique.

## Materials and methods

### In vivo* MRI data*

#### Myelin quantification: T1w/T2w maps

T1w/T2w structural datasets were downloaded from two distinct databases of the Human Connectome Project (HCP, http://www.humanconnectome.org): HCP (57 healthy subjects, aged 21–35) and HCP-Lifespan (10 healthy subjects, age 45–75). The reason behind adding ten more subjects from the latter dataset was to exclude any effect of age on T1w/T2w. HCP data was already preprocessed according to the HCP preprocessing pipeline (described in Van Essen et al. 2013), and made available also with the corresponding transformation fields to MNI space. HCP-Lifespan data were processed by the authors following the same processing pipeline as available at https://github.com/Washington-University/Pipelines.

Briefly, this preprocessing pipeline involves three distinct pipelines: (1) PreFreeSurfer pipeline, to generate gradient distortion corrected, bias field (B1 inhomogeneities) corrected T1w and T2w images, followed by brain extraction and registration to standard space; (2) FreeSurfer pipeline, to segment the structural volumes according to a specific parcellation, reconstruct white and pial cortical surfaces, and apply surface registration to FreeSurfer surface atlas; and, finally, (3) the PostFreeSurfer pipeline, which produces the final NIFTI files (including T1w/T2w maps) and GIFTI surface files registered to a surface atlas (Conte69), before downsampling and conversion to native space. After these steps, average surface cortical myelin maps were created using HCP Connectome Workbench (as described in Glasser and Van Essen 2011; Glasser et al. 2013). Similarly, average T1w/T2w MNI volumes for both HCP-Lifespan and HCP were obtained using the computed MNI transformations and FSL software package (fsl.fmrib.ox.ac.uk/fsl/fslwiki, Jenkinson et al. 2012).

#### Quantitative susceptibility mapping for iron content quantification

Data from 12 healthy subjects (70 ± 5 years, 6 female) were acquired on a 3 T MR Biograph Siemens scanner using a 12-channel coil. The protocol consisted in a spoiled gradient-echo sequence with flow compensation and the following parameters: TE = 19.6 ms, TR = 30 ms, flip angle = 12°, voxel size = 0.5 × 0.5 × 1 mm^3^, matrix size = 478 × 378, 160 slices. The Quantitative Susceptibility Maps (QSM) were obtained from phase images using the method described by Li et al. 2015. This approach includes the following steps: a sparse linear equation and least-squares algorithm-based method to derive an initial estimation of magnetic susceptibility; a fast-quantitative susceptibility mapping method to estimate the susceptibility boundaries and an iterative approach to estimate the susceptibility artifact from ill-conditioned k-space regions only. The procedure generates an unbiased estimate of tissue susceptibility with negligible streaking artifacts. QSM maps of each subject were spatially normalised to MNI template by applying T1-driven spatial non-rigid transformation, estimated with SPM12; QSM maps were voxel-wise averaged across all subjects to obtain a QSM average map.

#### Multi-echo T2 myelin water fraction atlas

An atlas of the average MWF distribution in the adult human brain has been made available at https://sourceforge.net/projects/myelin-water-atlas/ (Liu et al. 2019). In brief, this atlas was made using a multi-echo T2 approach, as described by MacKay et al. 1994, Zhang et al. 2015, using a 3D GRASE pulse sequence (Prasloski et al. 2012). This approach is currently one of the standard methods to quantify myelin water fraction using MRI, and it is expected to correlate with myelin density highly. In this study, we used this atlas to compare MWF values with T1w/T2w maps and histology in the callosal regions.

### Ex vivo *myelin quantification: histological staining*

#### Brain samples

We obtained four post-mortem brains at autopsy (with the prior informed consent of the person's relatives) from subjects without neurological or psychiatric diseases. To test whether we could replicate ex vivo the identical T1w/T2w distribution pattern in the corpus callosum across different ages, three brains (male, 68 years; female, 73 years; male, 82 years) from the Max Planck Institute for Human Cognitive and Brain Sciences were collected in Leipzig, Germany. One additional younger female brain (12 years) was obtained from King’s College Hospital, Department of Clinical Neuropathology, London, United Kingdom.

All samples were formalin-fixed at 4% for more than 4 weeks before manually dissecting the corpus callosum. The three older brain samples were cryoprotected in a 30% sucrose solution and sectioned with a freezing microtome (SM2000 R, Leica; Hyrax KS 34, Zeiss) in a sagittal plane at 25 μm. A similar procedure was followed for the young female brain where the sample was first formalin-fixed paraffin-embedded (FFPE) and then sectioned with a conventional microtome at 7 μm.

#### Myelin staining: luxol fast blue histology

The sections were mounted on coated slides and stained for myelin sheaths with an optimised Luxol Fast Blue (LFB) protocol (Mulisch and Welsch 2015). The deparaffinised sections were rinsed in distilled water for 5 min, and in 70, 85, and 96% alcohol for 3 min each. They were incubated overnight at a temperature of 60 °C in a 0,1% LFB solution (100 ml 96% alcohol, 0,1 g LFB (SERVA) and 0,5 ml 10% acetic acid), followed by 96% alcohol for 3 min, and distilled water for 5 min. They were subsequently rinsed in 0.05% lithium carbonate for 5 seconds, differentiated two times in 70% alcohol (30 s each time, with short movements to rinse out the excess colour), and rinsed twice in distilled water for 5 minutes. The sections were then dehydrated in graded alcohols, rinsed twice in Toluol and coverslipped with Entellan (Merck).

#### LFB optical density analysis

We digitized the sections of the three post-mortem older callosal samples with a digital camera (D90, Nikon), and the younger one with a photomicroscope with a motorized stage (Axio Imager M1, Zeiss; 4 × magnification). We estimated myelin density using an LFB Optical Density analysis approach (Beckmann et al. 2018) with the Nikon NIS-Elements Advanced Research 4.12 software. All acquisitions were made with the same light calibration. Three-channel RGB callosal images were converted to a single 256-grey scale. The 0–256 intensity range was rescaled from 1 to 7 and converted into a colour-coded map, where purple indicates the lowest level of myelin density and dark red the highest level of myelin density (Deshmukh et al. 2013).

### Definition of the callosal regions of interest (ROIs)

A sampling over 92 circular regions of interest (ROIs) was performed following a template of the midsagittal section of the corpus callosum. As shown in the results section, the template is organised according to the anatomical and geometrical subdivision proposed by Witelson 1989. It subdivides the corpus callosum into six macro-regions along the antero-posterior axis: rostrum/genu, rostral body, anterior midbody, posterior midbody, isthmus, and splenium. The template is specifically designed based on Witelson’s geometrical subdivision to take into account individual variability in callosal size among the subjects and allow comparisons among them (Witelson 1989).

### Statistical analysis

To quantitatively compare MRI maps with histological data, the same 92 ROIs sampling procedure was applied to the average T1w/T2w and MWF maps. Each map was sampled on two sagittal slices at + 4 and − 4 mm from the midsagittal section, and values were averaged. Histological LFB-OD data were averaged across the three adult callosal samples. Pearson correlations were performed to compare 92 ROIs LFB-OD vs T1w/T2w, LFB-OD vs MWF and finally T1w/T2w vs MWF.

## Results

**Fig. 1:**
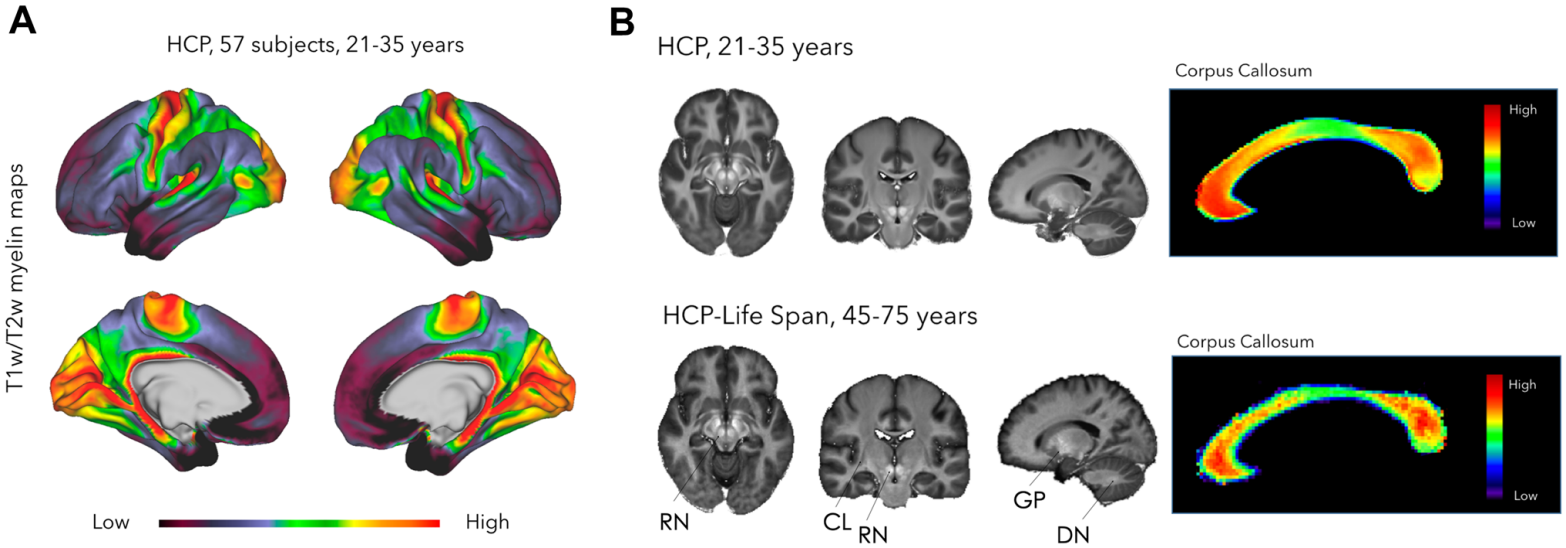
T1w/T2w myelin maps at cortical and callosal level. **A** T1w/T2w myelin maps at the cortical level of 57 healthy participants (aged 21–35) from the Human Connectome Project. T1w/T2w cortical maps show a gradient of T1w/T2w values from the primary motor (M1) and sensory areas (V1, S1, A1), which have higher values, to associative areas. This pattern is consistent with previous reports (Nieuwenhuys 2013; Van Essen and Glasser 2014; Nieuwenhuys et al. 2015). Colour scale: purple, low T1w/T2w values; red, high T1w/T2w values. **B** Left: averaged T1w/T2w maps of the whole brain of healthy participants from HCP (age 21–35, top) and HCP-Lifespan (age 45–75, bottom). The highest intensity signals are located in the red nuclei (RN) and globus pallidus (GP), while moderate and low intensities are in the dentate nucleus (DN) and claustrum (CL). Right: averaged T1w/T2w map of the corpus callosum of the same subjects. The colour-code map shows a heterogeneous distribution of T1w/T2w values in the different callosal subregions, with the highest T1w/T2w values in the rostrum/genu and slightly lower in the isthmus/splenium. Colour scale: purple, low T1w/T2w values; red, high T1w/T2w values

Figure 1A illustrates the cortical distribution of the T1w/T2w ratio in the 57 subjects from the HCP dataset. This result coherently replicates previous findings from Glasser and Van Essen (2011): high T1w/T2w ratio values can be found in regions with high cortical myelination, such as the primary motor-sensory and visual cortex. Figure 1B represents the volumetric distribution of T1w/T2w in the white matter for both the HCP and HCP-Life Span datasets. The highest T1w/T2w intensities is not in the white matter, but, instead, in grey matter regions such as the red nucleus, substantia nigra, globus pallidus and partially the dentate nucleus. This result is consistent in both the older and younger cohort. In the white matter of the corpus callosum, T1w/T2w maps reveal a heterogeneous distribution of values along the callosal subregions, with higher T1w/T2w values in the rostrum/genu and isthmus/splenium compared to the posterior midbody and the inferior splenium (Fig. 1B, right). This pattern was consistently observed in both the HCP and HCP-Life Span datasets, thus suggesting that this intensity heterogeneity is stable and not driven by age effects.

**Fig. 2:**
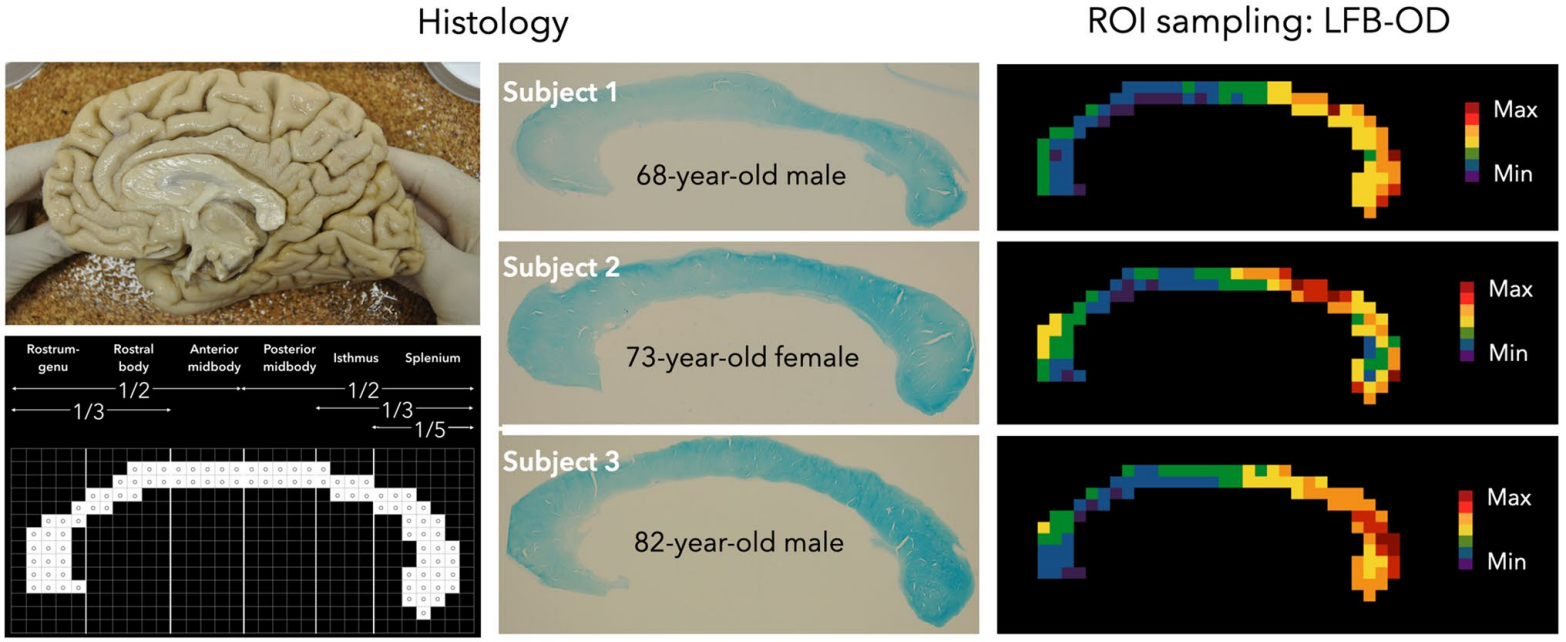
Histology and ROI sampling. Left, top: one of the four post-mortem brain samples with the corpus callosum in situ. Left, bottom: template of the corpus callosum showing 92 circular regions of interest (ROIs) based on the anatomical subdivision proposed by Witelson (1989). Values were calculated inside the circular ROIs in the centre of each of the 92 squares of the callosal template and then extended to the square surrounding the circle. Centre: Luxol Fast Blue (LFB)-stained midsagittal sections (25 µm thick) of the corpora callosa of subjects 1, 2 and 3. Right: ROI-based, colour-coded maps of the myelin distribution of subjects 1, 2 and 3 along the midsagittal section of the corpus callosum assessed ex vivo via histological myelin staining. The histological myelin distribution pattern of the corpus callosum is different from the T1w/T2w pattern. All three LFB callosal samples have low myelin density in the anterior part (mainly rostrum/genu) and higher myelin density in the posterior part (mainly isthmus/splenium). Colour scale: purple, low values; red, high values

**Fig. 3:**
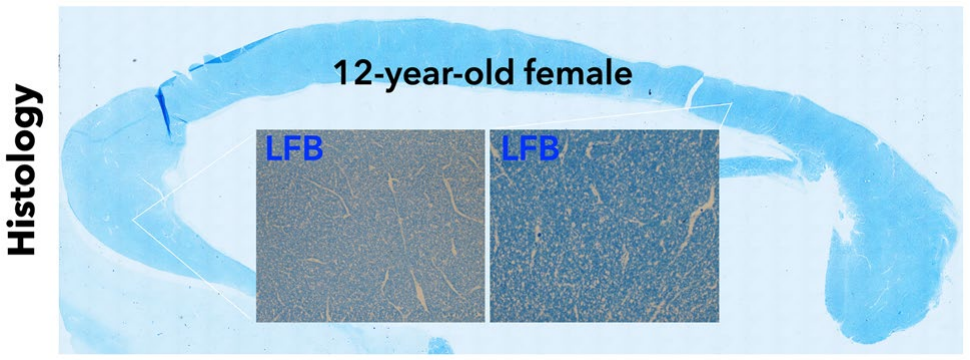
Myelin mapping of the corpus callosum: LFB-stained midsagittal section of the corpus callosum of a 12-year-old female. Insets: LFB myelin staining at higher magnification shows lower intensity in the anterior corpus callosum (rostrum/genu, left inset) and higher intensity in the posterior part (isthmus, right inset)

To test whether we could replicate ex vivo the identical T1w/T2w distribution pattern in the corpus callosum, we analysed three human post-mortem samples (ages: 68, 73, 82 years) stained with LFB. LFB staining can be seen in the histological slices (Fig. 2, centre) and with a 92 circular ROIs sampling approach defined along the midsagittal sections of the entire corpus callosum (Fig. 2, right). The histological myelin distribution in the corpus callosum derived from LFB-optical density analysis is different from the T1w/T2w pattern. In fact, in all three LFB callosal samples, myelin density in the anterior part (mainly rostrum/genu) is low and higher in the posterior part (mainly isthmus/splenium). This staining pattern is reproducible across a series of five consecutive sections for each case (see Supplementary Figs. 1–3). Indeed, the observed variance across the callosal regions was lower than the variance within the different samples. For each of the 92 callosal data points, we observed a normalised LFB intensity range extending from values of 1 to 7, with an average standard deviation across all regions of 1.47 but only an average standard deviation across samples of 0.62. An identical pattern, characterised by higher myelin content in the medium/posterior part of the corpus callosum, was also detected in the additional younger brain sample (female, age: 12 years), which was dissected and stained in a different laboratory (Fig. 3).

**Fig. 4:**
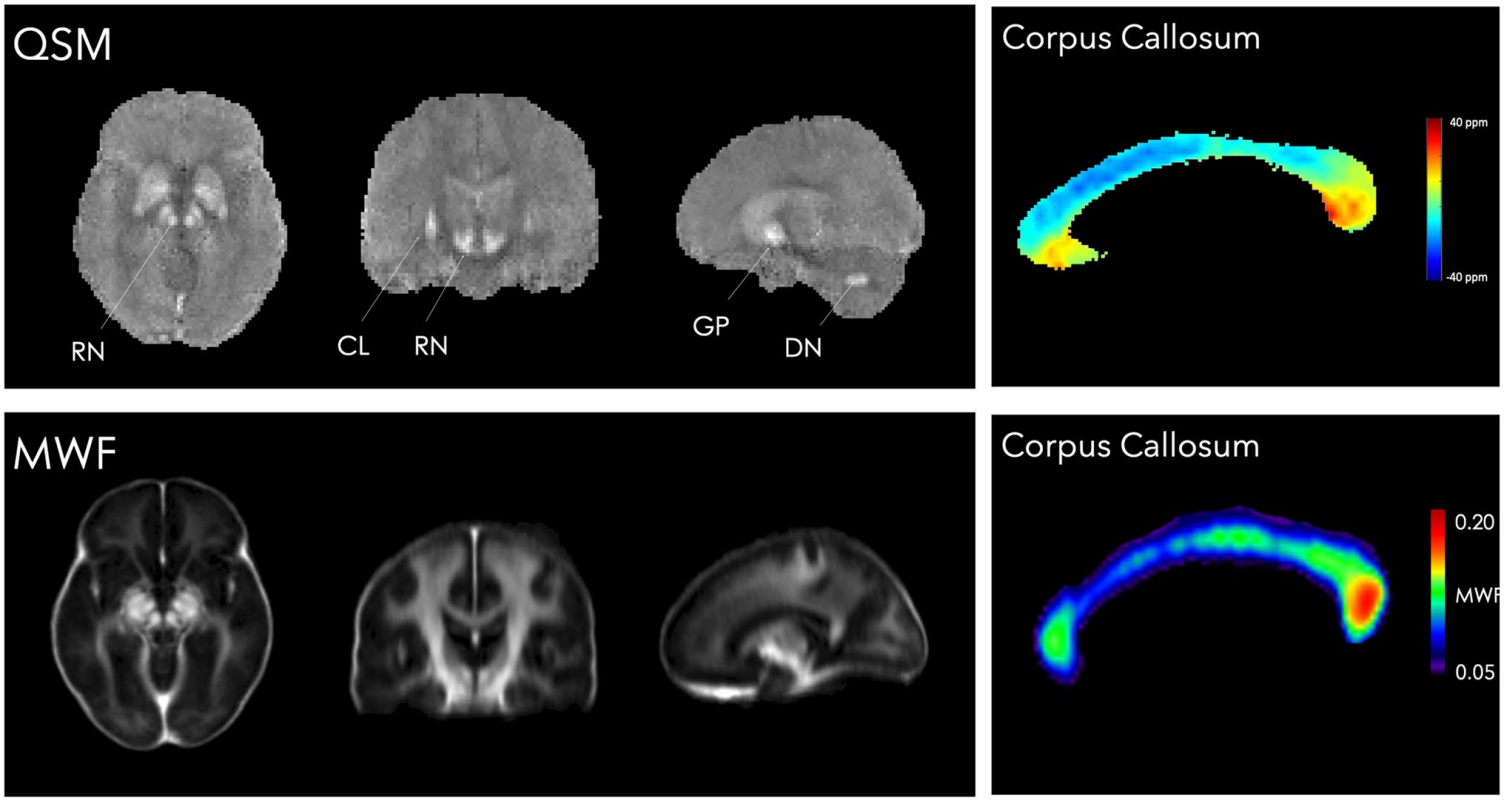
QSM and MWF cortical and myelin mapping. Left, top: average quantitative susceptibility maps (QSM) illustrating iron distribution in the whole brain in 12 healthy subjects (70 ± 5-year-old, 6 females). Higher intensities are localised in the red nuclei (RN) and globus pallidus (GP) as in the T1w/T2w maps. The high QSM intensity in the dentate nucleus (DN) and claustrum (CL) is not matched in the T1w/T2w maps represented in Fig. 1. Left, bottom: Myelin Water Fraction (MWF) maps from (Liu et al. 2019) averaged MWF atlas. Right: averaged QSM and MWF maps of the corpus callosum show different intensity patterns from anterior to posterior regions

To further examine whether T1w/T2w maps are modulated by iron content, we investigated the QSM map derived from an average of 12 healthy subjects (Fig. 4, top). As expected, regions with high QSM intensities are the red nucleus, substantia nigra and globus pallidus. These findings align well with what we found in the T1w/T2w maps. However, regions such as the dentate nucleus and claustrum have a high QSM intensity, but only moderate or low T1w/T2w values. At callosal level, QSM values are high in the inferior part of the genu, rostrum and inferior splenium, where T1w/T2w values are also high. However, the upper parts of the genu and splenium have a lower mean magnetic susceptibility, whereas the T1w/T2w intensity is higher. A discrepancy is also evident for the anterior midbody (QSM values very low, T1w/T2w medium-to-high), whereas the isthmus reveals relatively low intensities in both QSM and T1w/T2w maps.

Compared to the MWF atlas, T1w/T2w maps suggest a similar, increased intensity difference between white matter regions and cortical grey matter (Fig. 4, bottom). However, another pattern emerges when looking at differences within white matter regions. The internal capsule and the region corresponding to the cortico-spinal tract display a high content of MWF, whereas T1w/T2w has an opposite, darker contrast compared to more lateral white matter regions. By focusing on the body of the corpus callosum, the MWF atlas is characterised by relatively high myelination in the anterior genu, but lower intensities in the anterior body. Higher MWF intensities are evident from the posterior midbody and splenium, thus reproducing a pattern similar to the histological staining intensity.

**Fig. 5:**
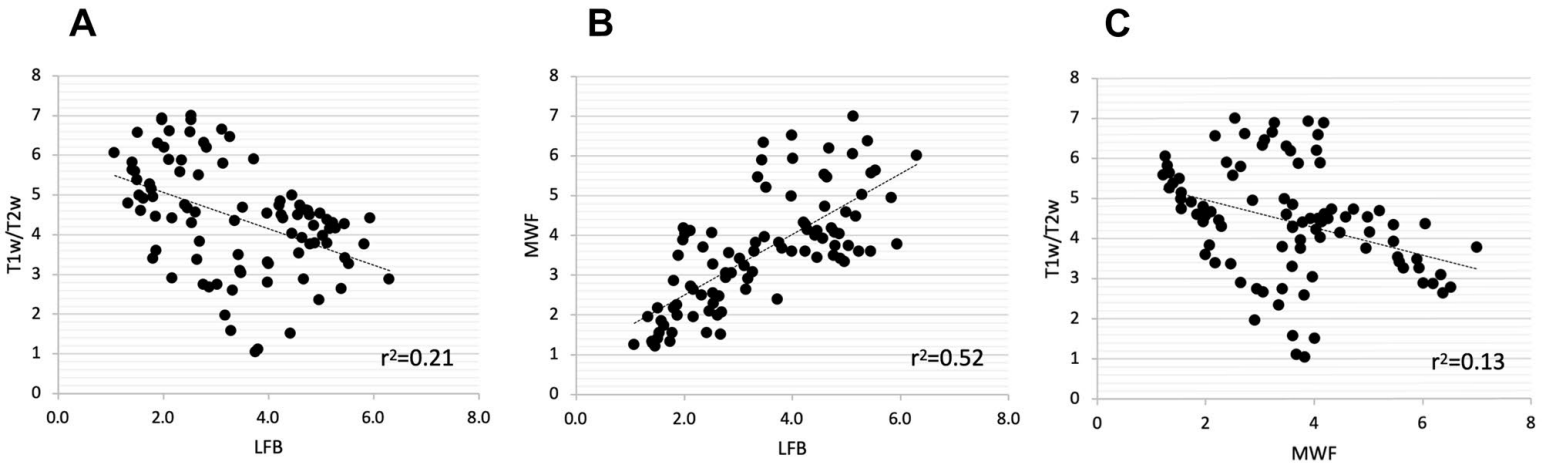
To quantitatively evaluate the correlation between T1w/T2w, MWF and histology, MRI maps were sampled using the same 92-ROI sampling scheme used for histology and correlated with the corresponding average LFB-OD values. Data is displayed on arbitrary units (from 1 to 7) for all methods. **A** T1w/T2w does not positively correlate with the LFB-Optical Density analysis but, instead, has a significant negative correlation (*r*^2^ = 0.21 *p* < 0.001). **B** MWF presents a strong and significant positive correlation with LFB (*r*^2^ = 0.52, *p* < 0.001), suggesting a good agreement between the two modalities. **C** T1w/T2w and MWF maps do not correlate positively, as they are characterised by a weak negative correlation (*r*^2^ = 0.13, *p* < 0.001)

To quantitatively evaluate the correlation between T1w/T2w, MWF and histology, we sampled the two MRI maps using the same 92-ROI sampling scheme used for histology. Figure 5 shows the correlation data: T1w/T2w does not positively correlate with LFB-Optical Density, but, instead, reveals a weak to moderate yet significant negative correlation (*r*^2^ = 0.21, *p* < 0.001, Fig. 5A). On the contrary, MWF is strongly and positively correlated with LFB (*r*^2^ = 0.52, *p* < 0.001, Fig. 5B), suggesting a good agreement between the two modalities. Finally, T1w/T2w and MWF maps are similarly weakly negatively correlated (*r*^2^ = 0.13, *p* < 0.001, Fig. 5C).

## Discussion

In this study, we applied multiple in vivo MRI and histological methods to clarify whether T1w/T2w is a method to map myelin in the white matter. Our results showed a discrepancy between T1w/T2w maps and the alternative imaging modalities we applied. While histological studies have confirmed that cortical regions with high T1w/T2w signal co-localise with high myelin levels (Glasser et al. 2014), this contrast does not correlate with alternative methods to quantify myelin in white matter regions. Overall, the T1w/T2w ratio reveals increased values in white matter regions compared to cortical grey matter, but, within white matter, its intensity variability does not follow the same pattern as illustrated by the other methods. Quite striking is the relatively low T1w/T2w intensity in the regions corresponding to the corticospinal tract and internal capsule compared to the other white matter regions with higher intensity. This pattern is not found in the MWF atlas, where the internal capsule and the corticospinal have a higher myelin content than white matter regions. When mapped against the callosal histology, T1w/T2w seems again to show a different pattern compared to LFB-OD analysis. In T1w/T2w, the genu is the region with the highest ‘myelination’, followed by the superior portion of the splenium. On the other hand, while indicating an increase in myelination in the most anterior part of the genu, LFB permits observing much stronger myelination in the posterior regions of the corpus callosum, which is consistent across all the histological samples and the subjects, irrespective of age and gender. This is further confirmed by the correlation analysis based on the 92-ROI sampling, where T1w/T2w does not positively correlate with the LFB nor the MWF measurements. On the contrary, MWF and LFB have a strong positive correlation (*r* = 0.72, *r*^2^ = 0.52, *p* < 0.001), thus supporting the idea that both modalities indeed capture myelin density. These observations suggest that the T1w/T2w contrast may not represent a suitable method for mapping the myelin in the corpus callosum and, arguably, in the white matter in general.

### Myelin and iron distribution

The discrepancy we found at the callosal level suggests that other biological factors should be considered when these maps are applied to the white matter. We adopted LFB as myelin staining, which is not only one of the most used staining methods globally and the reference staining tool used in routine diagnostics (Kluver and Barrera 1953; Lazzarini 2003), but it has also been widely used in previous papers to compare myelin mapping MRI methods quantitatively and to quantify changes in diseases or animal models (Laule et al. 2006; Khodanovich et al. 2017; Beckmann et al. 2018; Wood et al. 2016).

Luxol Fast Blue stain ‘highlights the blue myelinated axons of neurons in the white matter of the nervous system and the small dense round nuclei of oligodendrocytes that produce this myelin sheath’ (Lindberg and Lamps 2018). Myelin and iron distributions co-localise significantly in many regions (Ogg and Steen 1998; Fukunaga et al. 2010), especially in the visual cortex and in the motor/somatosensory cortex (Stüber et al. 2014), and prior MRI investigations of brain iron have been published (Gelman et al. 1999; Haacke et al. 2005). Therefore, a plausible alternative explanation is that T1w/T2w maps are modulated by, or might reflect, differences in iron density. Our results support this hypothesis, as very high T1w/T2w intensities were recorded in regions that are known for their high iron content, including the globus pallidus, substantia nigra and red nucleus (Rouault 2013; Piñero and Connor 2000, but see also Koeppen 1995). These high-intensity regions are evident in both the HCP-Lifespan dataset with older subjects and the younger group from HCP, a piece of evidence that seems to rule out any age effect.

Our QSM results confirm this overlap with iron at the level of these subcortical regions. However, this overlap is not present for other subcortical regions, such as the dentate nucleus and claustrum. Similarly, relatively high signal intensity is evident for both T1w/T2w and QSM maps in the inferior genu and the inferior splenium, but not in the superior genu, superior splenium and anterior midbody. In light of this, it seems appropriate to conclude that the iron content may modulate the T1w/T2w ratio only in some subcortical nuclei, and possibly some subregions of the corpus callosum, but it does not fully explain the origin of the T1w/T2w contrast. The lack of published histologically defined iron maps of the corpus callosum does not allow us to directly compare myelin vs. iron distribution to test further hypotheses beyond our QSM results.

MWF maps correlate well with histology and seem good myelin maps. However, various structures definitively known not to contain myelin also show up with high MWF, including the dura (especially the falx in the publicly available atlas), the cerebral arteries and veins, and the extraocular muscles. Moreover, MWF images seem strongly affected by iron (e.g., in the globus pallidus), similar to T1w/T2w, NODDI, T2w and quantitative T2. Overall, it can be intriguing to speculate on the potential differences in the iron presence (ferritin, within microglia, etc.) and if and how that may impact QSM and T1/T2 measures differently, although this is clearly outside the scope of the paper and will be clarified by future works.

### Myelin and axonal diameter

An alternative hypothesis is that the T1w/T2w signal might also be modulated by the complex distribution of different populations of fibres with varying axonal diameters. Histological maps of the axonal diameter distribution along the corpus callosum have been published by Aboitiz et al. (1992), and their pattern of distribution strikingly overlaps with our T1w/T2w maps. In particular, the higher T1w/T2w signal in the anterior part of the corpus callosum corresponds to a region containing small-diameter axons. In contrast, the central segment of the corpus callosum with a lower T1w/T2w signal corresponds to a region with predominantly large-diameter axons. Furthermore, the mixed low–high-low intensity pattern in the posterior corpus callosum parallels the large–small–large pattern of the axonal diameter distribution (Aboitiz et al. 1992).

Overall, at this point, we cannot exclude that the T1w/T2w signal is a rather aspecific contrast modulated by multiple biological factors. In fact, in addition to myelin, iron and fibre density, other glial cells, elements of extracellular space and vasculature may play a role. Additional research is needed to clarify the biological meaning and elucidate the T1w/T2w signal's complexity.

Other MRI methods have been proposed to quantify myelin mapping, such as multi-echo-T2 (MacKay et al. 1994; Zhang et al. 2015), mcDespot (Deoni et al. 2008; Deoni and Kolind 2015), quantitative T1 maps (Geyer et al. 2011; Geyer and Turner 2013; Bock et al. 2013), and macromolecular tissue volume (Mezer et al. 2013). All these methods attempt to provide a quantitative measure of myelin, and their histological validation is currently ongoing (Lazari and Lipp 2021; Laule et al. 2006; Laule 2008; Dula et al. 2010; Fatemi et al. 2011; Khodanovich et al. 2017). To date, whether myelin mapping biomarkers can give* absolute* and* quantitative* indications of myelin density is still an open question. Nevertheless, T1w/T2w maps cannot be fully considered quantitative because they heavily depend on the actual scanning parameters (i.e., multiple T1w and T2w contrasts can be obtained on the same subject by changing the MR acquisition parameters), and this effectively changes the final contrast and their reproducibility. Therefore, T1w/T2w maps should be assumed to provide qualitative information, and particular care should be taken when comparing results across studies or scanner manufacturers.

### Limitations of the study

This study has some limitations. Our group of older participants encompassed ten subjects. However, by comparing T1w/T2w maps in a younger cohort of 57 HCP participants, we obtained identical results, and we concluded that T1w/T2w findings are not age-dependent. Another limitation is that our report is based on an indirect comparison between in vivo and ex vivo maps acquired from different subjects. Ideally, MRI and histology should have been acquired from the same subjects. This is certainly feasible with animal models or by taking advantage of human post-mortem imaging, albeit for the latter the correspondence between in vivo T1w/T2w and post-mortem T1w/T2w still needs to be verified, and this would have introduced a further source of variability. Similarly, the QSM map and the MWF atlas are derived from different groups of individuals. However, as we detected the same T1w/T2w pattern for different cohorts and the same pattern in each histology sample, we expect that what is measured in this study is overall robust and beyond interindividual variability. Nevertheless, an approach that could be applied in future studies would be to collect QSM, multi-echo T2-MWF and T1w/T2w on the same group of subjects.

We adopted LFB as myelin staining for the reasons explained above, but we are aware that other histological methods are available, including staining for myelin basic protein (MBP) and myelin proteolipid protein (PLP). Moreover, a quantification of the callosal myelin density has been performed in the macaque brain using high-resolution Electron Microscopy (EM) data (Stikov et al. 2015a and b). However, while EM can provide accurate and precise quantification of myelin, only small sample regions can be selected. If samples and tissues are not homogenous, this may lead to notable sampling bias. Considering that our human samples were fixed by immersion, compared to perfusion fixation used in animal models, this may have introduced more local inhomogeneities, making the final interpretation of the results more difficult.

In this paper, we used the anatomical and geometrical subdivision of the corpus callosum proposed by Witelson (1989). We are aware that more refined parcellation subdivisions have been proposed, such as those by Hofer and Frahm (2006) and Caminiti et al. (2013), to study the precise topography of callosal projections. However, the choice of the Witelson subdivision was driven by the need to (i) compare our results with those obtained in the reference work by Aboitiz et al. (1992), (ii) define a reproducible geometrical constraint for the 92 regions of interest used to quantify myelin density across the whole corpus callosum, (iii) to provide comparable *anatomical* information with previous studies (Aboitiz et al. 1992; Zaidel and Iacoboni 2003; Catani and Thiebaut de Schotten 2012).

Finally, the handling and processing of post-mortem brains involved many steps that could potentially introduce biases. We cannot exclude that some of the inter-individual variabilities might be related to differences in slice location or inhomogeneity along the slice due to cutting artefacts or vascularization. However, these are unlikely to impact our findings as the same pattern of myelin density was preserved and commonly present in all the consecutive stained callosal slices, beyond inter-individual differences and across different laboratories (Supplementary Figs. 1–3).

Although the paper focuses on ‘putting to the test’ the T1w/T2w method as a myelin mapping tool, it is worth stating that all the imaging methods have strengths and weaknesses. For example, T1w/T2w maps’ strengths include ease of acquisition on 3 T scanners, high spatial resolution, high contrast-to-noise ratio (CNR), short acquisition times (Glasser and Van Essen 2011). Furthermore, T1w/T2w images are useful for other kinds of processing, including subcortical and cortical segmentation or surface generation (Glasser et al. 2014). At the same time, these are not quantitative, and there may be a second order anti-correlation with other white matter measures related to the predominant axonal diameter in the white matter.

Moreover, these are typically harder to acquire at the same acquisition resolution, field strength, acquisition time, and CNR as T1w/T2w. MWF images are closer to the histological profile, as shown here. Yet the publicly available datasets seem to have a very low spatial resolution, which, in turn, might lead to significant partial voluming and rather long acquisition time. MWF maps correlate well with histology. However, considering the non-myelinated structures displaying high MWF values, it is plausible that these are not entirely *specific* for myelin either. The quest for the most suitable MRI measure for quantifying myelin is still open (Mancini et al. 2020), and future studies can potentially extend this finding by using also MT-based methods (Hagiwara et al. 2018; Henkelman et al. 2001). On a more general note, methodological and performance variability, inconsistent reporting, publication bias and limited validation data available are reportedly some of the factors limiting the comparison between MRI-based myelin markers (Lazari and Lipp 2021; van der Weijden et al. 2021).

## Conclusions

Developing a reliable in vivo method for myelin mapping is crucial to understanding myelination in healthy and pathological conditions. This paper represents a preliminary attempt to clarify the biological underpinnings of the T1w/T2w signal. The results highlight important limitations in interpreting T1w/T2w as a valid myelin contrast in white matter. The discrepancy between T1w/T2w MRI maps, QSM, MWF and histological myelin maps suggests caution in using T1w/T2w as a white matter mapping method. Future studies are needed to clarify the exact biological meaning and the origin of the T1w/T2w signal in the white matter.

## Supplementary Information

Below is the link to the electronic supplementary material.

Supplementary file 1. Myelin mapping of the corpus callosum: additional Luxol Fast Blue-stained slices. We add additional stained sections to prove that the histology-based results are reproducible across a consecutive series of selected midsagittal sections. Supplementary Fig. 1 shows a consecutive series of LFB-stained sections of subject 1 (68-year-old male).

Supplementary file 2. Supplementary Fig. 2 shows a consecutive series of LFB-stained sections of subject 2 (73-year-old female).

Supplementary file 3. Supplementary Fig. 3 shows a consecutive series of LFB-stained sections of subject 3 (82-year-old male).

## Data Availability

This manuscript has no associated data.
