# Supplementary figures and images for: Mapping myelin in white matter with T1-weighted/T2-weighted maps: discrepancy with histology and other myelin MRI measures
Source: Brain Struct Funct. 2023 Jan 24;228(2):525–35. doi: 10.1007/s00429-022-02600-z (PMC9944377; doi:10.1007/s00429-022-02600-z)

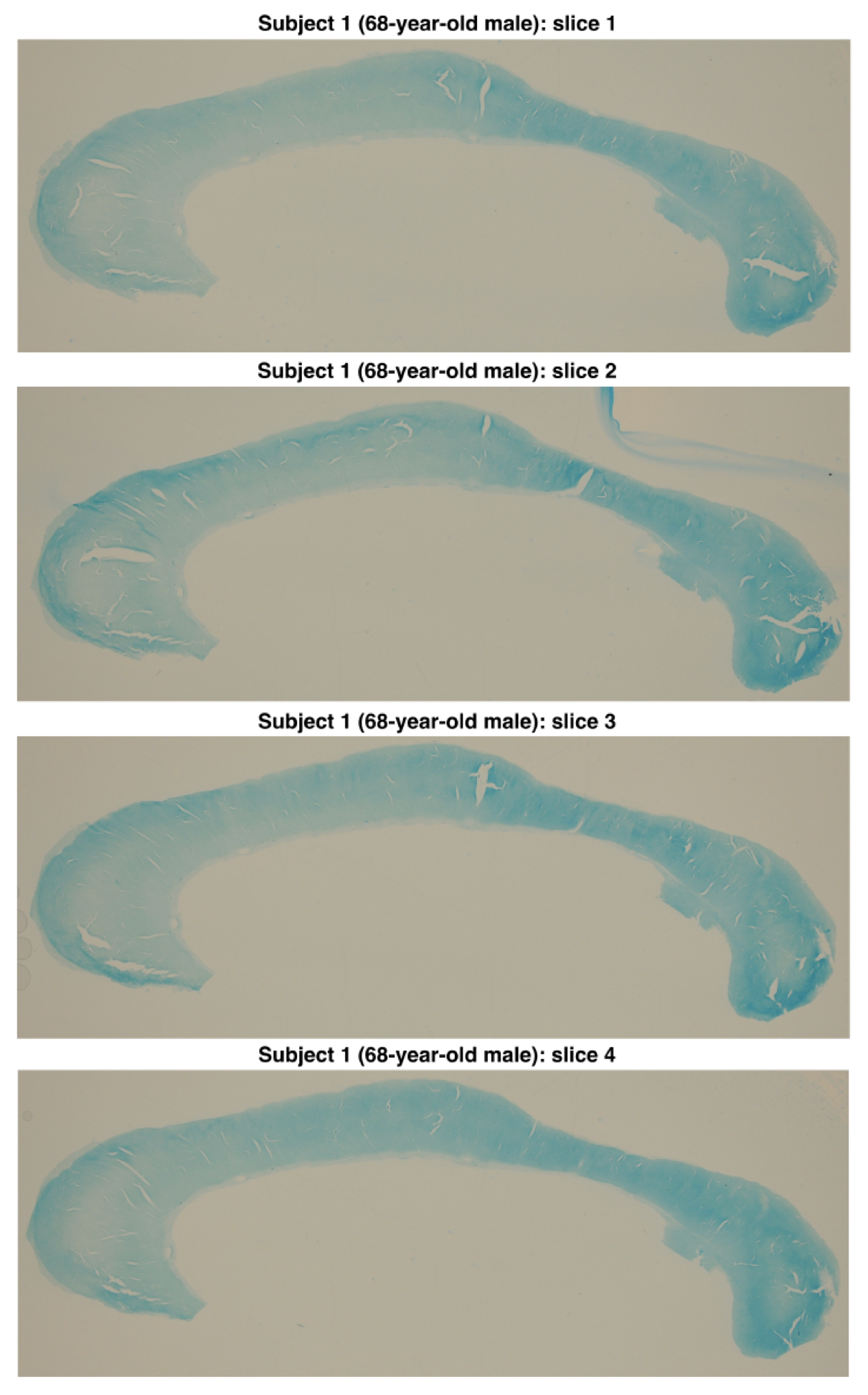

Supplement: Supplementary file 1 — Myelin mapping of the corpus callosum: additional Luxol Fast Blue-stained slices. We add additional stained sections to prove that the histology-based results are reproducible across a consecutive series of selected midsagittal sections. Supplementary Fig. 1 shows a consecutive series of LFB-stained sections of subject 1 (68-year-old male). [file 429_2022_2600_MOESM1_ESM.tiff]

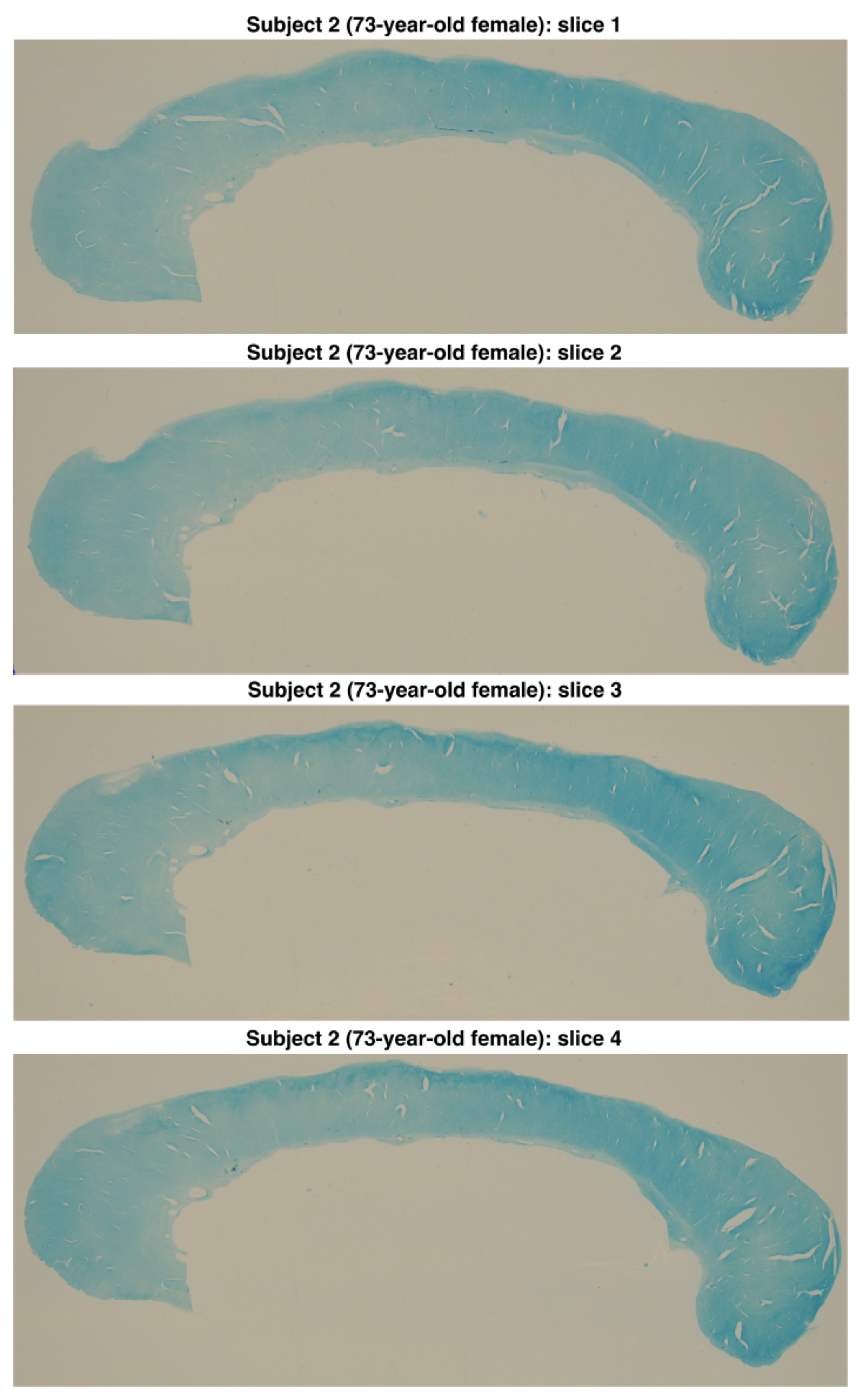

Supplement: Supplementary file 2 — Supplementary Fig. 2 shows a consecutive series of LFB-stained sections of subject 2 (73-year-old female). [file 429_2022_2600_MOESM2_ESM.tiff]

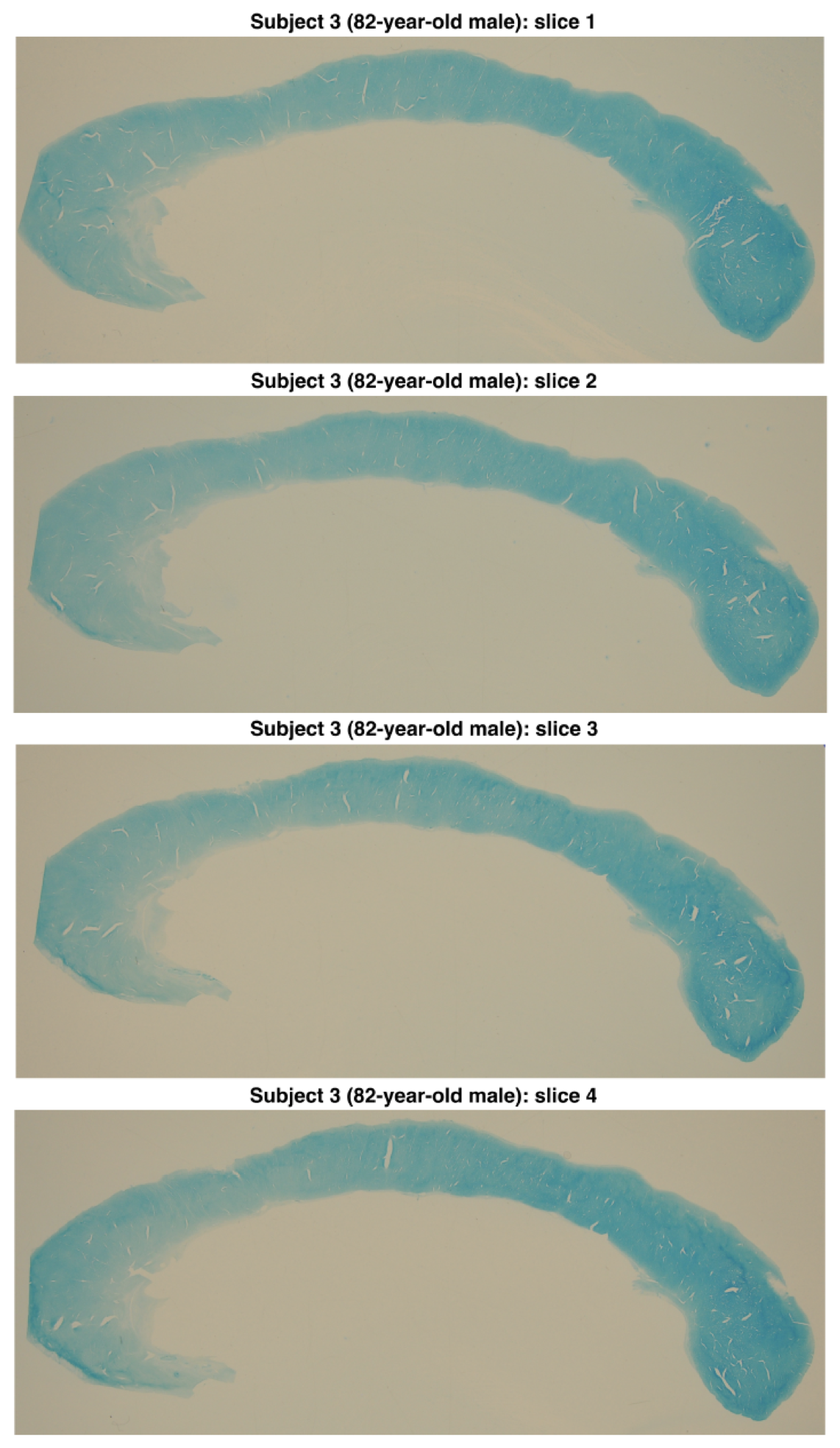

Supplement: Supplementary file 3 — Supplementary Fig. 3 shows a consecutive series of LFB-stained sections of subject 3 (82-year-old male). [file 429_2022_2600_MOESM3_ESM.tiff]
